# Cardiac and liver impairment on multiorgan MRI and risk of major adverse cardiovascular and liver events

**DOI:** 10.1038/s41591-025-03654-2

**Published:** 2025-05-07

**Authors:** Edward Jackson, Andrea Dennis, Naim Alkhouri, Niharika Samala, Raj Vuppalanchi, Arun J. Sanyal, Mark Muthiah, Rajarshi Banerjee, Amitava Banerjee

**Affiliations:** 1 Perspectum Ltd, Oxford, UK; 2 Arizona Liver Health, Phoenix, AZ USA; 3 Summit Clinical Research, San Antonio, AZ USA; 4 Indiana University School of Medicine, Bloomington, IN USA; 5 Virginia Commonwealth University, Richmond, VA USA; 6 Yong Loo Lin School of Medicine, National University of Singapore, Singapore, Singapore; 7 Division of Gastroenterology and Hepatology, Department of Medicine, National University Hospital, Singapore, Singapore; 8 Oxford University Hospitals NHS Trust, Oxford, UK; 9 University College London, London, UK

**Keywords:** Prognostic markers, Preventive medicine, Magnetic resonance imaging

## Abstract

Cardiovascular disease and metabolic dysfunction-associated steatotic liver disease are common conditions associated with high mortality and morbidity, yet opportunities for integrated prevention are underinvestigated. We explored the association between impairment in the liver (defined by increased iron-corrected T1 (cT1) time) and/or heart (reduced left ventricular ejection fraction ≤ 50) and risk of experiencing cardiovascular- or liver-related events or all-cause mortality among 28,841 UK Biobank participants who underwent magnetic resonance imaging. Using Cox proportional hazard models, adjusted for age, sex, body mass index, type 2 diabetes and dyslipidaemia, we observed that cardiac impairment was associated with increased incidence of cardiovascular events (hazard ratio (HR) 2.3 (1.9–2.7)) and hospitalization (HR 2.1 (1.8–2.4)). Liver impairment was associated with incident cardiovascular hospitalization (cT1 ≥ 800 ms, HR 1.3 (1.1–1.5)), liver events (cT1 ≥ 875 ms, HR 9.2 (3.2–26) and hospitalization (cT1 ≥ 875 ms, HR 5.5 (3.2–9.3). Associations between cT1 and liver events were maintained in participants with metabolic dysfunction-associated steatotic liver disease (*N* = 6,223). Reduced left ventricular ejection fraction (≤50) combined with elevated cT1 (≥800 ms) were associated with earlier cardiovascular events (time to event 0.8 versus 2.4 years; *P* < 0.05). Cardiac and liver impairment are independently, or in combination, associated with cardiovascular or liver events, suggesting a dual role for magnetic resonance imaging in integrated prevention pathways.

## Abstract (web summary)

In 28,841 individuals who underwent cardiac and liver MRI before being followed for a median of 4.3 years, detection of both liver and cardiac impairment by MRI was associated with cardiovascular- and liver-related hospitalization.

## Main

Cardiovascular diseases (CVDs) are the leading cause of global morbidity and mortality, attributable to modifiable, metabolic risk factors that continue to increase globally^1^. CVD prevention guidelines highlight the role of modifiable risk factors in risk prediction^2^. Metabolic dysfunction-associated steatotic liver disease (MASLD) (previously non-alcoholic fatty liver disease) is a term that describes steatotic liver disease associated with metabolic syndrome. It is defined by levels of elevated fat in the liver determined by imaging or biopsy and the presence of at least one of five cardiometabolic risk factors^3^. MASLD can progress to metabolic dysfunction-associated steatohepatitis (MASH), which is defined histologically by the presence of lobular inflammation and hepatocyte ballooning and is associated with a greater risk of fibrosis progression.

MASLD is a modifiable risk factor for CVD, as well as the leading global cause of chronic liver disease^4^. Up to 14% of those with MASLD may progress to more severe MASH with increasing fibrosis^5^, with increased risk of both major adverse cardiovascular and liver events; the latter include variceal hemorrhage, ascites and encephalopathy^6^, and hepatic cancer^7^ and liver transplantation^8,9^. The risk of cardiovascular (CV) outcomes increases progressively with worsening MASLD histology, independently of common cardiometabolic risk factors.

Early detection, treatment and prevention of major adverse CV and liver events are a priority in MASLD, particularly due to CV and hepatic therapeutic advances. However, drug development has focused on direct liver-related effects. Resmetirom^10^, the first therapeutic indication for MASH, received accelerated regulatory approval in 2024, and antiobesity drugs are also being considered^11,12^. Liver biopsy remains the mainstay of detection and prognostication in clinical trials for MASH, but limitations include high variability^13^ and complications. Integrated cardiac and liver risk assessment and alternative biomarkers to liver biopsy are needed for practical, reproducible risk prediction of endpoints in MASLD, for interventional and observational research and integrated care pathways. This creates a pressing need for non-invasive biomarkers that can stratify the increased risk of liver and CV outcomes due to liver impairment and investigation of how integrated imaging metrics of cardiac and liver impairment interact with regards to outcomes.

Echocardiography is often first choice for cardiac assessment, but cardiac magnetic resonance imaging is guideline-recommended^2,14^ for accurate assessment of structure and function. Cardiac and liver impairment on magnetic resonance imaging (MRI) are strongly associated^15^. Multiorgan imaging, particularly heart and liver, may help in stratifying the risk of CV and liver events in populations and patients with suspected MASLD. Liver assessment by quantitative MRI includes proton density fat fraction (PDFF) for fat content^16^ (recommended during Resmetirom treatment^17^) and iron-corrected T1-mapping (cT1) for inflammation^18,19^. Liver cT1 is associated with liver events in chronic liver disease^20^ and major CV events in the general population^21^, as cited in gastroenterology^22^, endocrinology^23^, hepatology^24,25^ and cardiology guidelines, which recommend quantitative MRI for CV risk stratification in MASLD^26^. Multiorgan MRI of the heart and liver is part of management of haemochromatosis and iron overload^27^ and may be relevant to other clinical pathways. In this prospective cohort study, we investigated associations between cardiac (left ventricular ejection fraction (LVEF)) and liver (cT1 and liver fat) impairment on MRI and (1) major adverse CV events and hospitalization, (2) major adverse liver events and hospitalization and (3) mortality (all-cause, CV and liver).

## Results

### Study population

**Fig. 1:**
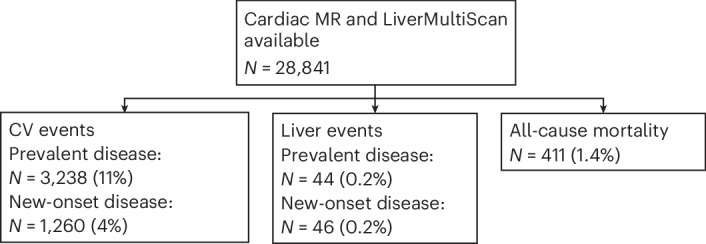
Participants from the UKB imaging data with available cardiac MR and LiverMultiScan data. Prevalent and incident occurrence of major CVD events, major liver events and all-cause mortality.

Of the study population, 28,841 had complete imaging data (Fig. 1). Median age was 64 (s.d. 8) years; 53% were female, and 17% had body mass index (BMI) ≥ 30. Median LVEF was 60%, cT1 was 693 ms and liver fat was 3% (Table 1). Median follow-up was 4.3 (interquartile range 3.7–5.2) years. Median time-to-event was 2.4 (interquartile range: 1.3–3.5) years (2.4 (1.2–3.6) years for CV events; 2.7 (1.5–3.7) years for liver events).

**Table 1:** Participant demographics for the whole cohort and divided by clinical outcomes

	Total cohort (*N* = 28,841)	Event free (*N* = 24,096)	CV event (*N* = 1,128)	Liver event (*N* = 46)	All-cause mortality (*N* = 398)	*P* value
Age (years)	64 (58–70)	63 (57–69)	68 (62–72)	68 (62–71)	71 (65–74)	7.6 × 10^−107^
Sex (% male)	13,571 (47%)	10,565 (44%)	688 (61%)	30 (65%)	252 (63%)	3.6 × 10^−41^
BMI (kg m^−^^2^)	25.8 (23.4–28.7)	25.6 (23.3–28.5)	26.5 (23.9–29.3)	27.6 (24.0–30.6)	26.7 (23.8–29.6)	6.2 × 10^−13^
Ethnicity (% white British)	26,356 (91%)	21,990 (91%)	1,045 (93%)	41 (89%)	363 (91%)	0.2
Current smoking (%)	932 (3.2%)	757 (3.1%)	47 (4.2%)	0 (0%)	19 (4.8%)	0.011
Daily alcohol intake (%)	4,684 (16%)	3,855 (16%)	202 (18%)	11 (24%)	85 (22%)	0.031
Waist circumference (cm)	87 (78–96)	86 (77–95)	91 (83–99)	94 (85–106)	91 (83–101)	1.5 × 10^−12^
Hypertension	4,821 (17%)	3,093 (13%)	277 (25%)	14 (30%)	122 (31%)	7.5 × 10^−51^
Type 2 diabetes	1,484 (5.1%)	994 (4.1%)	91 (8.1%)	11 (24%)	42 (11%)	3.8 × 10^−18^
Dyslipidemia	6,197 (21%)	4,255 (18%)	314 (28%)	19 (41%)	137 (34%)	2.1 × 10^−34^
cT1 (ms)	693 (662–729)	690 (660–727)	704 (670–739)	728 (692–772)	712 (675–754)	1.6 × 10^−27^
Liver fat (%)	3.1 (2.2–5.4)	3.0 (2.2–5.2)	3.5 (2.4–6.6)	3.6 (2.8–6.9)	3.2 (2.3–5.9)	1.1 × 10^−11^
Liver iron (mg Fe per g)	1.21 (1.12–1.35)	1.21 (1.12–1.34)	1.22 (1.12–1.36)	1.16 (1.11–1.35)	1.22 (1.11–1.36)	0.36
LVEF (%)	59.8 (55.9–63.7)	59.9 (56.2–63.8)	58.8 (54.7–63.1)	58.5 (53.5–61.9)	58.4 (54.1–62.7)	4.2 × 10^−16^

*P* value represents the outcomes of two-sided Kruskal–Wallace tests (for continuous variables) or the Chi-squared test (for binary variables) to test for significant differences across the four groups, without adjustments made for multiple comparisons.

During follow-up, there were (*n* (incidence per 1,000 person-years)): 1,260 (11) CV events, 1,711 (14), CV hospitalizations; 46 (0.35) liver events and 311 (2.4) liver hospitalizations; and 411 (3.2) all-cause deaths. The most common CV events were coronary artery disease (*n* = 494), atrial fibrillation (*n* = 386) and then stroke (*n* = 162) (Supplementary Tables 2 and 3). The most common liver events were cirrhosis (*n* = 9), portal hypertension (*n* = 8), hepatocellular carcinoma (*n* = 7) and liver failure (*n* = 7) (Supplementary Tables 4 and 5). The most common cause of all-cause mortality was CV (*n* = 61); there were six recorded liver-related deaths (Supplementary Table 6). Individuals with CV, liver or mortality events were older (*P* < 0.001), had higher BMI (*P* < 0.001) and were more likely to be male (*P* < 0.001) than those without any events (Table 1).

In those with MASLD (mean age 64 (s.d. 7) years, 43% were female and 42% BMI ≥ 30 kg m^−2^), average cT1 was 748 ms, liver fat was 11%, and LVEF was 60%. There were 319 (13 per 1,000 person-years) CV events, 445 (16) CV hospitalizations; 12 (0.4) liver events, 131 (4.7) liver hospitalizations; and 93 deaths (3.3). Eighteen (19%) (0.6) were related to CV hospitalization, of which 13 (14%) (0.5) were specifically CV event related and 4 (4%) (0.1) were liver related.

In those with cT1 and liver fat at two imaging timepoints (*n* = 2,325), average time between scans was 2.3 years. At their second visit, follow-up participants were similar in age, sex and BMI to the whole UK Biobank (UKB) with imaging metrics available at their first imaging visit (Supplementary Table 7). In the time following the second imaging visit, there were 61 new CV events, 89 CV hospitalizations, one liver event, 20 liver hospitalizations and 16 deaths (two CV related, zero liver related).

### Major adverse CV events and hospitalization

**Fig. 2:**
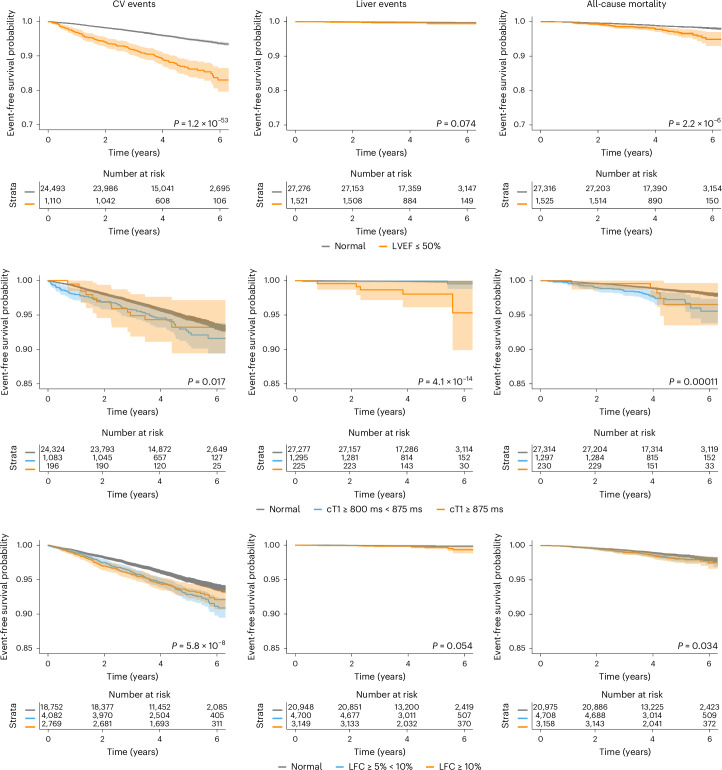
Kaplan–Meier plots for survival of clinical events stratified by reduced LVEF, elevated liver cT1 and liver fat content. Plots show survival from CV events (left), liver events (middle) and all-cause mortality (right). Shaded areas represent the 95% CIs for the survival curve. Log-rank test was used to assess differences in rates of events between groups. Two-sided *P* value < 0.05 was considered statistically significant.

Reduced LVEF (≤50%) was associated with CV events (hazard ratio (HR) 2.3, 95% confidence interval (CI) 1.9–2.7) and hospitalization (2.1, 1.8–2.4). cT1, as a continuous variable, was associated with CV events (1.1, 1.0–1.2) and hospitalizations (1.1, 1.1–1.2). cT1 > 800 ms was associated with CV hospitalization (1.3, 1.1–1.5) (Table 2 and Fig. 2). Elevated liver fat (≥5%) was associated with neither CV events (1.0, 1.0–1.1) nor hospitalization (1.0, 0.9–1.2).

**Table 2:** Association between MRI metrics of liver health (cT1 and liver fat) and cardiac function (LVEF) and risk of CV and liver events, hospitalization and all-cause mortality

	Major CV events	CV-related hospitalization	Major liver events	Liver-related hospitalization	All-cause mortality
**Cox proportional HR (95% CI) (*n*** = **28,841)**					
LVEF ≤ 50% (*n* = 1,525)	**2.3 (1.9–2.7)**	**2.1 (1.8, 2.4)**	1.7 (0.6, 4.2)	0.7 (0.4, 1.3)	**1.5 (1.1, 2.1)**
LVEF > 50% and cT1 ≥ 800 ms (*n* = 1,431)	1.0 (0.8–1.3)	**1.3 (1.0, 1.5)**	1.9 (0.7, 4.9)	**3.5 (2.6, 4.8)**	**1.7 (1.2, 2.4)**
LVEF ≤ 50% and cT1 < 800 ms (*n* = 1,429)	**2.3 (1.9–2.7)**	**2.1 (1.8, 2.4)**	1.6 (0.6, 4.6)	0.8 (0.5, 1.5)	**1.5 (1.1, 2.1)**
LVEF ≤ 50% and cT1 ≥ 800 ms (*n* = 96)	**2.2 (1.2–4.2)**	**2.3 (1.4, 3.7)**	3.7 (0.5, 27.8)	1.7 (0.4, 6.8)	**2.5 (1.0, 5.7)**
cT1 continuous	**1.1 (1.0–1.2)**	**1.1 (1.1, 1.2)**	**1.7 (1.3, 2.2)**	**1.6 (1.4, 1.7)**	**1.2 (1.1, 1.4)**
cT1 800–875 ms (*n* = 1,297)	1.0 (0.8, 1.3)	**1.3 (1.1, 1.5)**	0.7 (0.2, 3.0)	**3.1 (2.2, 4.3)**	**1.7 (1.2, 2.4)**
cT1 ≥ 875 ms (*n* = 230)	1.1 (0.6, 1.9)	1.1 (0.7, 1.8)	**9.2 (3.2, 26)**	**5.5 (3.2, 9.3)**	1.6 (0.6, 3.8)
Liver fat (continuous, per s.d.)	1.0 (1.0, 1.1)	1.0 (1.0, 1.1)	1.1 (0.8, 1.4)	**1.4 (1.3, 1.6)**	1.0 (0.9, 1.1)
Liver fat 5–10% (*n* = 4,706)	1.1 (0.9, 1.2)	1.1 (0.9, 1.2)	0.5 (0.2, 1.4)	**1.7 (1.3, 2.4)**	1.0 (0.8, 1.3)
Liver fat ≥10% (*n* = 3,158)	1.0 (0.9, 1.2)	1.0 (0.9, 1.2)	1.2 (0.5, 2.7)	**3.2, (2.3, 4.3)**	1.0 (0.8, 1.4)
**MASLD subset (*n*** = **6,223)**					
cT1 continuous	1.0 **(**0.9, 1.1)	1.1 **(**1.0, 1.2)	**1.8** (**1.2, 3.0)**	**1.4** (**1.3, 1.7)**	1.2 (1.0, 1.4)
cT1 800–875 (*n* = 941)	0.9 (0.6, 1.2)	1.2 (0.9, 1.5)	1.0 (0.2, 5.2)	**2.2** (**1.5, 3.4)**	1.4 (0.8, 2.4)
cT1 ≥ 875 ms (*n* = 184)	0.9 (0.4, 1.8)	0.8 (0.4, 1.5)	**8.7** (**2.0, 39)**	**3.8** (**2.1, 7.0)**	1.8 (0.6, 4.9)
Liver fat continuous (per s.d.)	1.0 (0.9, 1.1)	1.0 (0.9, 1.1)	1.4 (1.0, 2.0)	**1.4** (**1.2, 1.5)**	1.0 (0.8, 1.2)
Liver fat ≥10% (*n* = 2,508)	1.0 (0.8, 1.2)	0.9 (0.8, 1.1)	2.2 (0.6, 7.5)	**1.7** (**1.2, 2.4)**	1.0 (0.7, 1.6)

HR (Cox proportional hazard ratio adjusted for age, sex, BMI, type 2 diabetes and dyslipidemia) with 95% CIs are reported. Statistically significant results (two-sided *P* value < 0.05) are shown in bold. Results are shown for the whole cohort and the MASLD subset.

**Fig. 3:**
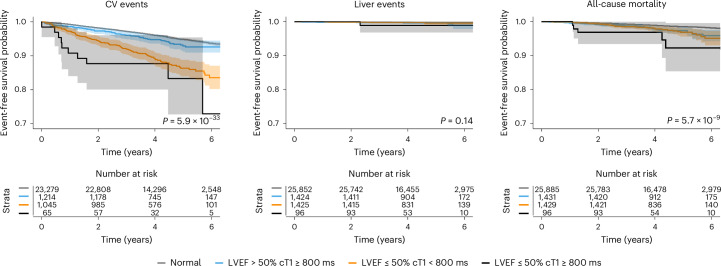
Kaplan–Meier plots for survival of clinical events stratified by combinations of elevated cT1 and decreased LVEF. Plots show survival from CV events (left), liver events (middle) and all-cause mortality (right). Shaded areas represent the 95% CIs for the survival curve. Log-rank test was used to assess differences in rates of events between groups. Two-sided *P* value < 0.05 was considered statistically significant.

Reduced LVEF (<50%) and cT1 ≥ 800 ms were associated with both CV events (2.2, 1.2–4.2) and hospitalization (2.3, 1.4–3.7) (Table 2) and shorter time to CV events than those without this combination of reduced LVEF and increased cT1 (0.78 versus 2.4 years; *P* < 0.05) (Fig. 3). In those with MASLD, there was no association between liver impairment on MRI and CV events or hospitalization. Reduced LVEF was associated with CV events and hospitalization. Reduced LVEF was associated with individual CV events: coronary artery disease (HR 1.8, 1.4–2.3, adjusted for age, sex and BMI only), myocardial infarction (1.8, 1.2–2.7), atrial fibrillation (2.7, 2.1–3.4), heart failure (6.7, 5.1–9.3) and stroke (1.9, 1.2–2.9). cT1 as a continuous variable was associated with coronary artery disease (1.2, 1.1–1.3) and atrial fibrillation (1.1, 1.0–1.2). Liver fat ≥5% was also associated with coronary artery disease (1.2, 1.0–1.4) and myocardial infarction (1.6, 1.2–2.2). Reduced LVEF with increased cT1 ≥ 800 ms was associated with further increased risk of atrial fibrillation (HR 4.6, 2.5–8.4) (Supplementary Table 8).

In those with repeat LiverMultiScan (*n* = 2,325, mean follow-up time = 3 years (2.9–3.1)), increasing cT1 was associated with increased CV events and hospitalizations (combined: HR 2.1, 1.1–3.7, *P* < 0.01) compared with stable cT1, with no corresponding association for increasing PDFF.

### Major adverse liver events and hospitalization

Reduced LVEF was not associated with liver events or hospitalization. Continuous cT1 was associated with higher rates of liver events (1.7 (1.3, 2.2)) and hospitalization (1.6 (1.4, 1.7)). Liver fat content (LFC) was associated with increased liver hospitalizations (1.4, 1.3–1.6) but not liver events (1.1, 0.8–1.4). The cT1 ≥ 800 ms group had increased liver hospitalization (3.1, 2.2–4.3), and cT1 ≥ 875 ms had increased liver events (9.2, 3.2–26) (Table 2). Liver fat ≥5% was associated with liver hospitalization only (1.7, 1.3–2.4), as was liver fat ≥10% (3.2, 2.3–4.3).

In those with MASLD, cT1 ≥ 875 ms was associated with liver events (8.7, 2.0–39) and liver hospitalization (3.8, 2.1–7.0). Liver fat ≥10% was associated with liver hospitalization only (1.7, 1.2–2.4).

### Mortality

Reduced LVEF was associated with all-cause mortality (1.5, 1.1–2.1). As a continuous variable, cT1 was associated with all-cause mortality (1.2, 1.1–1.4), unlike elevated LFC (1.0, 0.9–1.1). Reduced LVEF with elevated cT1 (≥800 ms) had an HR of (2.5, 1.0–5.7) even after exclusion of deaths post-COVID-19 hospitalization HR (2.9, 1.2–7.0). This persisted in the population with prevalent CVD (Supplementary Table 9a).

There were 80 CV-related deaths, of which 61 were related to major CV events. Elevated cT1 was associated with CV mortality (2.4, 1.2–4.6), but elevated liver fat was not (0.6, 0.3–1.0). Elevated cT1 with normal LVEF, reduced LVEF with normal cT1 and a combination of reduced LVEF and elevated cT1 were all associated with increased CV mortality (2.5, 1.2–5.0; 3.5, 1.9–6.2; and 6.4,1.9–21.4, respectively) (Fig. 3 and Supplementary Table 8).

Due to low event rates (six liver deaths), age, sex and BMI adjustment were not possible. Of the six events, one had reduced LVEF, three had cT1 ≥ 800 ms (none with cT1 ≥ 875 ms), and five had liver fat above 5% (two had ≥10%).

## Discussion

In the largest prospective analysis of cardiac and liver imaging and cardiac and liver events to date, we report three findings. First, impairment in heart or liver is associated with increased risk of major adverse CV events, and combined cardiac and liver impairment was associated with accelerated time to major CV event. Second, liver impairment from cT1, but not LFC, is associated with major adverse liver events. As liver cT1 increases, risk of liver events surpasses risk of CV events. Third, liver impairment by cT1 was associated with increased risk of all-cause, CV and liver mortality.

Risk prediction tools^28^ and clinical pathways do not routinely include MASLD or liver disease more broadly as a risk factor, despite associations with CVD comparable to other risk factors, such as chronic kidney disease and type 2 diabetes, leading to substantial variation in risk management in people with MASLD^29^. Prior systematic reviews have shown that CV risk is increased in the context of MASLD, particularly MASLD^30,31^. In our analyses, major CV events were much more common than major liver events (11 versus 0.38 cases per 1,000 person-years) even in those with MASLD. Both cardiac (reduced LVEF) and liver impairment (cT1 and PDFF) and worsening liver impairment (cT1) were associated with CVD, corroborating a recent cohort study from Taiwan^32^. A recent systematic review and meta-analysis confirmed that lean and non-lean individuals with MASLD have similarly increased risk of CVD^33^. Therefore, full screening for CV risk and disease is indicated to reduce morbidity and mortality in people with and at risk of MASLD, as recommended by American Heart Association^26^. As shown in Supplementary Table 1, there are signs of cardiac abnormality in the population with cT1 > 800 ms and preserved ejection fraction, such as elevated ventricular mass and wall thickness.

Liver impairment, by assessment of inflammation (cT1) on MRI, was strongly associated with major adverse liver events: cT1 > 875 ms was linked with nine-fold increased risk. This association was absent with liver fat >10% and reduced ejection fraction, even in those with MASLD. Our results demonstrate a superiority of cT1 over liver fat as a monitoring measurement for individuals with MASLD, likely owing to the positive linear relationship observed between cT1 and increasing liver fibrosis^34^ as oppose to liver fat, which decreases with advancing fibrosis^35,36^. Patients, practitioners and policymakers would prefer surrogate endpoints for clinical outcomes as alternatives to invasive liver biopsy, whether to reduce procedure-associated morbidity, healthcare utilization or cost. Although we do not directly compare with liver biopsy in this study, cT1 is likely to be a suitable alternative in routine clinical practice and in phase 2b and 3 clinical trials for MASLD.

Overall, we show that cardiac and liver impairment on MRI are associated with increased all-cause mortality, but although increased CV mortality was associated with both cardiac and liver impairment, increased liver mortality seemed more related to liver impairment. Similar trends were seen for CV and liver hospitalizations. As noted above, cT1 > 800 ms represents the transition between MASLD and MASH. This transition is defined on histology by the presence of ballooning, inflammation and increased fibrosis. As such, elevated cT1 represents a level of underlying liver impairment that drives both the increased risk of liver outcomes and also risk of CVD and mortality. Recent research has identified that two distinct genetic subtypes of MASLD may exist that determine the likely risk of clinical trajectory toward liver-related or CV-related outcomes^37^, supporting a multiorgan approach for risk stratification and onward clinical management.

A recent Japanese study suggested that cardiometabolic targets may be applicable to individuals with MASLD for prevention and treatment of CVD but found that predictors of liver events and mortality were lacking^38^. We show that rates of liver hospitalization rise to the level of CV events with increasing cT1 (Tables 3 and 4), which has previously been shown to be positively associated with histological levels of inflammation and fibrosis^34^. Therefore, there may be increased disease activity that drives fibrogenesis, as higher liver-related events are expected with advancing fibrosis stages^6^.

**Table 3:** Incidence of events by combined LVEF and cT1 category

New-onset events	*N* at risk	LVEF > 50% and cT1 < 800 ms (*n* = 25,885)	LVEF > 50% and cT1 ≥ 800 ms (*n* = 1,431)	LVEF ≤ 50% and cT1 < 800 ms (*n* = 1,429)	LVEF ≤ 50% and cT1 ≥ 800 ms (*n* = 96)
Hypertension	24,020	779 (3.6%)	71 (7.2%)	63 (5.8%)	4 (7.3%)
Type 2 diabetes	27,356	63 (0.3%)	22 (1.9%)	8 (0.6%)	1 (1.4%)
Liver hospitalization	28,638	234 (0.9%)	64 (4.6%)	11 (0.8%)	2 (2.1%)
Major liver events	28,797	35 (0.1%)	6 (0.4%)	4 (0.3%)	1 (1.0%)
Major CVD events	25,603	1,052 (4.5%)	71 (5.8%)	127 (12%)	10 (15%)
CVD hospitalization	28,841	1,375 (5.3%)	122 (8.5%)	197 (14%)	17 (18%)
All-cause mortality	28,841	333 (1.3%)	36 (2.5%)	37 (2.6%)	5 (5.2%)

*N* represents the total proportion of participants in each group. *N* at risk shows number in whole cohort remaining after those with prevalent disease are removed from analysis. *N* at risk shows number in whole cohort remaining after those with prevalent disease are removed from analysis.

**Table 4:** Incidence of events by cT1 category

New-onset events	*N* at risk	cT1 < 800 ms (*n* = 27,314)	cT1 800–875 ms (*n* = 1,297)	cT1 ≥ 875 ms (*n* = 230)
Hypertension	24,020	842 (3.7%)	68 (7.7%)	7 (4.4%)
Type 2 diabetes	27,356	71 (0.3%)	18 (1.7%)	5 (2.9%)
Liver events	28,797	39 (0.1%)	2 (0.2%)	5 (2.2%)
Liver hospitalization	28,638	245 (0.9%)	50 (3.9%)	16 (7.3%)
Major CVD events	25,603	1,179 (4.8%)	69 (6.4%)	12 (6.1%)
CVD hospitalization	28,841	1,572 (5.8%)	122 (9.4%)	17 (7.4%)
All-cause mortality	28,841	370 (1.4%)	36 (2.8%)	5 (2.2%)

*N* represents the total proportion of participants in each group. *N* at risk shows number in whole cohort remaining after those with prevalent disease are removed from analysis. *N* at risk shows number in whole cohort remaining after those with prevalent disease are removed from analysis.

Our study has several clinical and public health implications. First, there is a major potential value of cT1 in the monitoring individuals with MASLD for risk of major CV and liver events, with opportunities for prevention and treatment. Second, based on the cumulative increase in CV risk when cardiac and liver impairment are evident on MRI, there is value in cardiac MR being performed in MASLD and liver MRI in patients with CVD. Third, our results highlight the potential benefit of integrating care pathways and guidelines for metabolic multiorgan disease, as already in place for haemochromatosis and iron overload^27^. Fourth, consideration of metabolic risk factors and diseases, whether at a local, national or international level, should consider the important overlaps between cardiac and liver disease, from risk factors through to mortality^39^.

There are also research implications for our findings. First, there is clearly a need for greater efforts and resources to be devoted to the pathophysiologic mechanisms through to the therapy for integrated CV and liver diseases, which, to date, have been seen, treated and researched in separate silos. Second, therapeutic studies should urgently use and evaluate liver impairment by cT1 as a surrogate endpoint for liver and CV outcomes. Third, further research should consider the exact value of cT1 and liver disease in risk prediction and its potential incorporation in clinically useable tools, alongside health economic consideration of which at-risk populations would benefit the most from a multiorgan stratification approach. Previous research has suggested that detecting risk of major CVD or liver-related events in people with preexisting risk factors is cost effective owing to the likely decrease in downstream health costs^40,41^. This is particularly relevant in the current climate of rapid development of antiobesity medicines, where the benefit of multiorgan screening in high-risk patients with metabolic syndrome, type 2 diabetes or prevalent CVD could be used to guide treatment decisions. Fourth, further research should identify further differences by subtype of CVD and MASLD at a national population scale.

### Limitations

A major strength of this analysis is access to the large samples the UKB affords, including the large subgroup with MASLD and extensive history and follow-up data. However, UKB is 90% white, so, as with all UKB studies, caution should be exercised before translating these research findings to non-white populations. There are ongoing studies collecting multiorgan MR metrics of the heart and liver and outcomes that will allow investigation of the high-risk phenotypes identified in this study in more diverse populations.

UK Imaging Diabetes Study Seeing Diabetes Clearly (UKIDS, https://clinicaltrials.gov/study/NCT05057403) is an ongoing prospective, observational cohort study in adult patients with type 2 diabetes lacking history of CVD, which contains a diverse range of ethnicities. The Dallas Heart and Minds study is currently collecting liver and cardiac MR imaging metrics and outcomes in a non-UK population.

Although the sample size of the study is large, the follow-up period is relatively short (median follow-up time 4 years), and the number of events, especially liver events, is relatively small. This limits the statistical power of the study and its ability to determine longer-term outcomes. As the UKB is an ongoing, long-term study that continues to collect and update clinical outcomes and continues to enroll new participants into the imaging substudy, we hope that future analysis will be able to investigate the longer-term implications of these findings.

A further limitation in this study is that we have not included hepatic encephalopathy (HE) as part of major liver events. In previous liver-specific studies with histology, HE was coded prospectively. This is not feasible in a general population without known liver disease. In those histology-specific studies, liver fat was not a predictor of HE, but fibrosis was, so it is likely that this study underestimates the predictive nature of cT1 as it may under-report major liver events through HE. HE hospitalizations (coded in the UKB under ‘K76.8: Other specified diseases of liver’) have been captured under liver hospitalizations, but mortality and new events have not been specifically captured. Follow-on work should include independent validation and comparison with liver biopsy as more detailed follow-up access for electronic health records. In addition, further exploration into more advanced cardiac MR metrics, such as strain and aortic flow, is warranted to further understand the potential to risk stratify to CV and liver events.

## Conclusions

Impairment of the heart and liver on multiorgan MRI is associated with major adverse CV events, and liver inflammation is associated with major liver events. We highlight the need for holistic multiorgan assessment in the management of people at risk of cardiometabolic conditions, such as early heart failure and MASLD, and demonstrate the value of quantitative MRI in this assessment to identify treatable disease.

## Methods

### Study design and participants

This prospective cohort study involved UKB participants enrolled in the imaging substudy between January 2016 and February 2020 (ref. ^42^). Individuals aged 40–69 years old were invited for MRI examination (heart and abdomen), including quantitative mapping, as previously described^43^. UKB has approval from the North West Multi-Centre Research Ethics Committee and obtained written informed consent from all participants before the study. Data were extracted under access application 9914. Those with complete imaging data for LVEF %, liver inflammation (cT1, ms) and LFC (%) were followed up for clinical outcomes, as specified prospectively by UKB.

### Assessments

Participants were scanned at one of the UKB imaging centers on Siemens Aera 1.5T scanners (Syngo MR D13) at baseline. A subset of participants were invited back for a follow-up LiverMultiscan if the baseline scan was of sufficient image quality and they lived within a certain proximity to an imaging center^44^. Cardiac imaging involved a combination of several gated cine series^45^. Liver imaging was performed using the LiverMultiScan protocol (Perspectum Ltd) in the UKB abdominal imaging protocol. Image analysis and further cardiac MRI metrics are available (Supplementary Table 1).

### Outcomes

Events (major adverse CV and liver), hospitalization (CV and liver) and mortality (all-cause, CV and liver) were determined based on ICD-10 coding as previously reported^21,46^ and detailed in the Supplementary Information section ‘Outcomes Measures’.

### Statistical analysis

Rates of new-onset events were calculated as the number of events during follow-up divided by the number of person-years at risk among patients who did not have the condition at enrollment and were reported as events per 1,000 person-years. Associations between imaging metrics and incident major adverse events, hospitalization and mortality were assessed in Cox proportional hazard models, adjusting for age, sex, BMI, prevalent dyslipidaemia and prevalent type 2 diabetes and reported as HRs with 95% CIs. Analysis of individual CV events were adjusted for age, sex and BMI only. Baseline was considered the day of attending the first imaging visit. Continuous variables were reported following *Z*-score normalization and categorized using predefined, clinical thresholds. These were ejection fraction below 50%, cT1 800–875 ms and cT1 ≥ 875 ms and liver fat 5–10% and ≥10%. Thresholds for cT1 are based on transition from MASLD to MASH to high-risk MASH and for liver fat between steatosis and severe steatosis (Supplementary Information section ‘Imaging Thresholds’). There was no correcting for multiple comparisons. Results are reported as point estimates with 95% CIs; the CIs have not been adjusted for multiplicity, so the intervals should not be used to infer definitive associations.

Unadjusted cumulative probability of events by groups defined by thresholds are represented by Kaplan–Meier curves. Three separate subgroup analyses included individuals with MASLD (defined as LFC > 5%), presence of at least one cardiometabolic risk factor (based on biometrics, diabetes diagnosis and blood biomarkers) and excluding those with high alcohol consumption (based on self-reported alcohol intake frequency)^3^ and those with and without prevalent CVD. Further subgroup analysis was conducted in those with LiverMultiScan data at two imaging timepoints to study change over time. As data collection occurred during and after the COVID-19 pandemic, a sensitivity analysis of all-cause mortality was performed, excluding those never hospitalized for COVID-19 (as a primary or contributory cause of death). Statistical analysis was performed using R software (v.4.0.4, in RStudio v.2024.12.0), with a *P* value < 0.05 considered statistically significant.

### Ethics approval

UKB data use (project application no. 9914) was approved by the UKB according to their established access procedures. UKB has approval from the North West Multi-Centre Research Ethics Committee (MREC) as a Research Tissue Bank (RTB) approval. This approval means that researchers do not require separate ethical clearance and can operate under the RTB approval.

### Reporting summary

Further information on research design is available in the Nature Portfolio Reporting Summary linked to this article.

## Online content

Any methods, additional references, Nature Portfolio reporting summaries, source data, extended data, supplementary information, acknowledgements, peer review information; details of author contributions and competing interests; and statements of data and code availability are available at 10.1038/s41591-025-03654-2.

## Supplementary information

Supplementary Information Supplementary methods, Tables 1–10 and Fig. 1.

Reporting Summary

## Data Availability

All data in this study is from the UKB. UKB data are made available to researchers from research institutions with genuine research inquiries, following IRB and UKB approval. Requests to access these datasets should be directed to access@ukbiobank.ac.uk. Code for generating results in this paper will be made available via the UKB returns system.
