# Supplementary material for: Cardiac and liver impairment on multiorgan MRI and risk of major adverse cardiovascular and liver events
Source: Nat Med. 2025 May 7;31(7):2289–96. doi: 10.1038/s41591-025-03654-2 (PMC12283365; doi:10.1038/s41591-025-03654-2)
Supplement: Supplementary file 1 — Supplementary methods, Tables 1–10 and Fig. 1. [file 41591_2025_3654_MOESM1_ESM.pdf]

nature medicine

Supplementary information https://doi.org/10.1038/s41591-025-03654-2

Cardiac and liver impairment on multiorgan MRI and risk of major adverse cardiovascular and liver events

In the format provided by the authors and unedited

Cardiac and liver impairment on multi-organ MRI and risk of major adverse cardiovascular and liver events

Contents

Image analysis	2
Outcomes measures	2
Imaging thresholds	4
Repeat imaging	5
Missing Data	5
Table S1: CMR Metrics of UKBB	5
Table S2: Prevalence and incidence of CVD events	6
Table S3: Prevalence and incidence of CVD hospitalisations	7
Table S4: Prevalence and incidence of major liver events	7
Table S5: Prevalence and incidence of major liver related hospitalisation	8
Table S7: Demographics of UKBB with and without follow-up scan	9
Table S8: Risk of individual cardiac events by LVEF, cT1 and liver fat	10
Table S9a: Association between MRI metrics of liver health (cT1 and liver fat) and cardiac function (LVEF) and risk of cardiovascular and liver events, hospitalization and all-cause mortality in those with prevalent CVD	11
Table S9b: Association between MRI metrics of liver health (cT1 and liver fat) and cardiac function (LVEF) and risk of cardiovascular and liver events, hospitalization and all-cause mortality in those without prevalent CVD	12
Table S10a: Association between MRI metrics of liver health (cT1 and liver fat) and cardiac function (LVEF) and risk of cardiovascular and liver events, hospitalization and all-cause mortality in males	13
Table S10b: Association between MRI metrics of liver health (cT1 and liver fat) and cardiac function (LVEF) and risk of cardiovascular and liver events, hospitalization and all-cause mortality in females	14
Supplementary Figure 1a: Forrest plot of hazard ratios by cT1 and ejection fraction category	15
Supplementary Figure 1b: Forrest plot of hazard ratios by cT1 threshold	16
References	16

Image analysis

Experienced cardiac MRI analysts used CVI42v5.11 (Cardiovascular Imaging Inc, Canada) to trace manually the myocardium in the end-diastolic and end-systolic phases in each of the short-axis views to calculate ventricular function and to estimate ejection fraction (EF); Liver MRI data was analysed automatically and reviewed by trained analysts using the LiverMultiScan® software to generate PDFF and cT1.

Outcomes measures

Sex Sex is acquired from central registry at recruitment, but in some cases updated by the participant. Hence this field may contain a mixture of the sex the NHS had recorded for the participant and self-reported sex.

Major adverse cardiovascular (CV) events as defined in Bosco et al., 2021 and listed in (2), reproduced here.

**Name**	**ICD-10 Codes**
Ischaemic Heart Disease + Coronary Artery Disease	I20, I24, I25
Myocardial Infarction	I21, I22, I23
Atrial Fibrillation	I48
Venous thromboembolism	I74, I80, I82
Heart Failure	I50
Stroke	I60, I61, I62, I63, I64, I69

Major adverse liver events: Based on ICD-10 codes from Shang et al, 2024, (3))

**Name**	**ICD-10 Codes**
Liver cirrhosis	K74.6
Oesophageal Varices	I85.0 I98.3, I98.2
Gastric Varices	I86.4
Hepatorenal Syndrome	K76.7
Ascites excluding cancer (except HCC) and/or heart failure diagnosis	R18.9 AND excluding C00-C21, C23-C99, I50, I09.0, I09.9, I11.0, I13.0
Portal Hypertension	K76.6
Hepatocellular Carcinoma	C22.0
Liver Failure	K72.1, K72.9
Liver Transplant	Z94.4

Hepatic encephalopathy (K76.82) has previously been reported as the most common major adverse liver event in patients with MASLD. In the UKBB this is only available as part of K76.8 (other diseases of liver). This group is included in the liver hospitalisation assessment but not as a specific liver event.

Hospitalisation

Cardiovascular and liver-related were recorded, including individuals admitted to hospital and occupying a hospital bed, including both admissions where an overnight stay was planned and day cases. Cardiovascular hospitalisation was defined as in (2) as 'primarily circulatory hospitalisation' and is defined as any new-onset event contained in Chapter IX of the ICD-10 code list in UK biobank field 41202. Liver hospitalisation was taken as any new-onset event containing ICD-10 codes K74, K75 or K76 from UK biobank field 41271.

Prevalent Dyslipidaemia

Prevalent dyslipidaemia is based either on clinically elevated levels of cholesterol or triglycerides taken at a participant's baseline visit, or a clinical diagnosis of dyslipidaemia (ICD-10 code E78 Disorders of lipoprotein metabolism and other lipidaemia's).

Type 2 diabetes

Prevalent type 2 diabetes is taken from a recorded diagnosis of type 2 diabetes prior to imaging visit, based on fields 130708 (non-insulin-dependent diabetes mellitus) and 130714 (unspecified diabetes mellitus).

Prevalent CVD

Prevalent CVD is prevalent hypertension (UKBB field ID 131286 essential (primary) hypertension) or previous MACE.

Imaging thresholds

Cardiac

- Left ventricular ejection fraction (LVEF) was estimated from CMR with a threshold of LVEF < 50% used to indicate reduced ejection fraction (4).

Liver

- Iron-corrected cT1: <800ms - healthy, 800-875ms - above upper limit of normal, and ≥875ms-higher risk of NASH with fibrosis (5).
- Liver fat <5%- healthy, 5-10%- simple steatosis, and ≥10%- severe steatosis (6).

cT1 clinical thresholds are based on the relationship between iron-corrected T1 measurements of the liver and histology. They are linked to key transitions in the progression from MASLD to high-risk MASH and have also been related to liver related outcomes in previous smaller studies. cT1 is recommended as a marker of fibroinflammatory change in guidelines for diagnosis and management of MASLD. The clinical threshold of 800 ms is considered the upper limit of normal and is the recommended threshold to identify those in transition from simple steatosis to and cT1 values ≥ 875 ms are associated with a disease activity and significant fibrosis in high-risk MASH patients (7). Thresholds for levels of liver fat are linked to histological levels of steatosis. 5-10%- simple steatosis, and ≥10%- severe steatosis (6).

Repeat imaging

In the subset of participants with two imaging timepoints and clinical outcomes data, progressors on imaging with those with follow-up value was greater than the metrics published repeatability coefficients. That was an increase of 46ms for cT1(8), and an absolute increase of 1.6% for PDFF (9).

Missing Data

Any participants missing cT1, PDFF or LVEF were excluded from the study. A small number of participants had missing BMI data taken at their imaging visit. These missing values were imputed based on BMI taken at baseline and first follow-up visit, using methods from Roca-Fernandez et al. 2023 (2). There were no missing data for age or sex. To account for different cutoff dates within UKBB follow-up data, all follow-up data was right censored after the earliest cutoff date of October 2022.

Table S1: CMR Metrics of UKBB

In this table p-value indicates result of two-sided Mann-Whitney U test to determine where CMR metrics in the LVEF > 50% & cT1≥800ms are different from the LVEF > 50% & cT1<800ms group. .

CMR Metrics	LVEF > 50% & cT1<800ms (n = 25,885)	LVEF > 50% & cT1≥800ms (n = 1,431)	LVEF≤ 50% & cT1<800ms (n = 1,429)	LVEF ≤ 50% & cT1≥800ms (n = 96)	p-value
**Left ventricular ejection fraction (%)**	60.2 (56.6, 63.9)	60.2 (56.5, 64.1)	47.4 (44.3, 49.0)	47.2 (41.6, 48.9)	0.80
**Left ventricular end diastolic volume (ml)**	141 (122, 165)	146 (124, 168)	169 (144, 195)	168 (144, 196)	6.5e-04
**Left ventricular end systolic volume (ml)**	56 (46, 68)	58 (46, 69)	90 (76, 106)	89 (76, 104)	0.03
**Left ventricular stroke volume (ml)**	85 (74, 99)	88 (75, 100)	78 (65, 90)	77 (66, 89)	3.4e-04
**Left ventricular circumferential strain (%)**	-22.6 (-24.7, -20.7)	-22.4 (-24.5, -20.2)	-16.5 (-18.1, -14.4)	-15.8 (-17.7, -13.0)	0.01
**Left ventricular longitudinal strain (%)**	-18.62 (-20.34, -17.00)	-18.41 (-20.07, -16.77)	-15.24 (-16.96, -13.34)	-14.65 (-16.33, -12.45)	0.00
**Left atrial ejection fraction (%)**	61 (57, 67)	61 (56, 66)	56 (48, 61)	55 (37, 61)	0.01
**Left atrial stroke volume (ml)**	42 (35, 50)	44 (36, 52)	40 (32, 49)	40 (31, 47)	4.8e-05

CMR Metrics	LVEF > 50% & cT1<800ms (n = 25,885)	LVEF > 50% & cT1≥800ms (n = 1,431)	LVEF≤ 50% & cT1<800ms (n = 1,429)	LVEF ≤ 50% & cT1≥800ms (n = 96)	p-value
**Left ventricular myocardial mass (g)**	80 (67, 98)	93 (78, 109)	100 (86, 116)	106 (92, 123)	1.0e-70
**Left ventricular myocardial wall thickness (mm)**	5.55 (5.06, 6.11)	6.15 (5.60, 6.69)	6.12 (5.63, 6.62)	6.41 (5.88, 7.01)	2.7e-141
**Right ventricular ejection fraction (%)**	58.0 (54.2, 61.6)	57.4 (53.9, 61.2)	50.0 (45.3, 54.1)	49.5 (43.2, 53.8)	0.00
**Right ventricular end diastolic volume (ml)**	150 (128, 178)	155 (130, 180)	169 (141, 194)	165 (147, 190)	7.2e-04
**Right ventricular end systolic volume (ml)**	63 (51, 78)	66 (53, 80)	85 (70, 101)	83 (72, 96)	1.3e-04
**Right ventricular stroke volume (ml)**	87 (75, 102)	89 (75, 103)	84 (69, 98)	83 (68, 95)	0.02
**Ascending aortic distensibility (10^-3/mmHg)**	1.46 (0.98, 2.24)	1.55 (1.03, 2.21)	1.33 (0.92, 2.14)	1.51 (1.12, 2.03)	0.03
**Descending aortic distensibility (10^-3/mmHg)**	2.12 (1.50, 2.94)	2.04 (1.51, 2.68)	1.85 (1.29, 2.66)	1.86 (1.32, 2.22)	0.00

Table S2: Prevalence and incidence of CVD events

	ICD-10 Codes	N (new onset)	N (prevalent)
**Coronary Artery Disease**	I20, I24, I25	494	1528
**Myocardial Infarction**	I21, I22, I23	72	90
**Atrial Fibrillation**	I48	386	649
**VTE**	I74, I80, I82	107	624
**Heart Failure**	I50	39	28
**Stroke**	I60, I61, I62, I63, I64, I69	162	319
**Total**		1260	3238

Table S3: Prevalence and incidence of CVD hospitalisations

ICD-10 Codes	N (new onset)	N (prevalent)
**Any new-onset primarily circulatory hospitalization, I00-90**	1947	N/A

Table S4: Prevalence and incidence of major liver events

	ICD-10 Codes	N (new onset)	N (prevalent)
**Liver cirrhosis**	K74.6	9	11
**Esophageal Varices**	I85.0 I98.3, I98.2	3	8
**Gastric Varices**	I86.4	5	2
**Hepatorenal Syndrome**	K76.7	1	0
**Ascites**	R18.9 AND excluding C00-C21, C23-C99, I50, I09.0, I09.9, I11.0, I13.0	6	19
**Portal Hypertension**	K76.6	8	4
**Hepatocellular Carcinoma**	C22.0	7	0
**Liver Failure**	K72.1, K72.9	7	0
**Liver Transplant**	Z94.4	0	0
**Total**		46	44

Table S5: Prevalence and incidence of major liver related hospitalisation

	ICD-10 Codes	N (new onset)	N (prevalent)
**Fibrosis and cirrhosis of liver**	K74	17	15
**Other inflammatory liver diseases**	K75	16	23
**Other diseases of liver**	K76	278	165
**Total**		311	203

Table S6: Incidence of mortality

	N
**CVD events (Includes IHD and MI)**	80
**Pancreatic Cancer**	35
**Lung Cancer**	24
**COVID-19**	21
**Prostate cancer**	16
**Colorectal Cancer**	6
**External cause/injury**	8
**Breast Cancer**	6
**Dementia**	4
**Emphysema**	3
**Liver event (primary or secondary cause of death)**	**6**
**Other**	202
**Total**	411

Table S7: Demographics of UKBB with and without follow-up scan

Demographic	No follow up, N = 31,272[1]	Follow-up, N = 2,325[1]
**Age at first imaging visit**	65 (58-70)	63 (57-68)
**Age at second imaging visit**		65 (59-71)
**Sex (% Male)**	14,805 (47%)	1,126 (48%)
**Ethnicity (% white British)**	28,379 (91%)	2,180 (94%)
**BMI at first imaging visit (kg/m^2)**	25.9 (23.6-28.7)	25.7 (23.6-28.2)
**BMI at second imaging visit (kg/m^2)**		25.5 (23.3-28.2)

[1]Follow-up data was analysed on a set of participants with full LiverMultiScan metrics at first and second UKBB imaging visits.

Table S8: Risk of individual cardiac events by LVEF, cT1 and liver fat

	CAD	MI	AF	VTE	HF	Stroke	CV related Mortality
**Full cohort N = 28,841**							
**LVEF ≤50% (n = 1525)**	**1.8 (1.4-2.3)**	**1.7 (1.2-2.6)**	**2.8 (2.3-3.6)**	1.4 (0.8-2.6)	**7.0 (5.2-9.3)**	**1.9 (1.3-2.9)**	**3.3 (1.9, 5.6)**
**LVEF > 50% and cT1 ≥ 800ms (n = 1431)**	1.2, (0.9-1.7)	1.1 (0.7-1.9)	1.0 (0.7-1.4)	0.9 (0.4-1.9)	0.9 (0.4-1.6)	0.9 (0.5-1.8)	**2.5 (1.2-5.0)**
**LVEF ≤ 50% and cT1 < 800ms (n = 1429)**	**1.8 (1.4-2.3)**	**1.8 (1.2-2.7)**	**2.7 (2.1-3.4)**	1.5 (0.8-2.8)	**6.7 (5.1-9.3)**	**1.9 (1.2-2.9)**	**3.5 (1.9-6.2**
**LVEF ≤50% and cT1 ≥800ms (n = 96)**	1.9 (0.8-4.5)	1.0 (0.1-7.2)	**4.6 (2.5-8.4)**	NA	**6.2 (2.7-14.3)**	2.1 (0.5-8.6)	**6.4 (1.9 – 21.4)**
**cT1 (continuous, per s.d.)**	**1.2 (1.1-1.3)**	1.1 (1.0-1.3)	**1.1 (1.0-1.2)**	1.0 (0.9-1.2)	1.1 (1.0-1.3)	1.0 (0.9-1.1)	**1.4 (1.1, 1.7)**
**cT1 800-875ms (n = 1297)**	1.3 (0.9-1.7)	1.1 (0.7-1.9)	1.2 (0.9-1.6)	0.7 (0.3-1.6)	1.1 (0.7-1.8)	1.1 (0.6-1.9)	**2.4 (1.2, 4.6)**
**cT1 ≥875ms (n = 230)**	0.9 (0.4-2.3)	0.5 (0.1-3.6)	1.2 (0.6-2.6)	1.9 (0.6-6.1)	0.7 (0.2-2.9)	0.5 (0.1-3.7)	2.5 (0.6, 10.6)
**Liver fat (continuous, per s.d.)**	1.1 (1.0-1.1)	1.0 (0.9-1.2)	1.0 (0.9-1.2)	0.9 (0.8-1.1)	0.8 (0.7-1.0)	1.0 (0.8-1.1)	1.0 (0.8, 1.2)
**Liver fat 5-10% (n = 4706)**	**1.2(1.0-1.4)**	**1.6 (1.2-2.2)**	0.8 (0.7-1.0)	0.8 (0.5-1.2)	1.0 (0.8-1.4)	1.1 (0.8-1.5)	0.5 (0.2, 0.9)
**Liver fat ≥10% (n = 3158)**	1.2 (0.9-1.5)	1.1 (0.7-1.6)	1.0 (0.8-1.2)	0.6 (0.3-1.0)	0.7 (0.5-1.0)	0.8 (0.5-1.2)	0.9 (0.5, 1.7)

Cox proportional hazard ratios for individual cardiac events. Values shows are the multivariate adjusted Cox proportional hazard ratio (95% CI), adjusted for age, sex and BMI. CV-related mortality is adjusted for age, sex, BMI dyslipidaemia status and prevalent type 2 diabetes. Statistically significant results ($p<0.05$) are shown in bold.

Table S9a: Association between MRI metrics of liver health (cT1 and liver fat) and cardiac function (LVEF) and risk of cardiovascular and liver events, hospitalization and all-cause mortality in those with prevalent CVD

	Major CV events	CV-related hospitalisation	Major liver events	Liver-related hospitalisation	All-cause mortality
**Cox proportional hazard ratio (95% CI), N = 6,692**					
LVEF ≤50% (n = 604)	**2.9 (2.1, 4.0)**	**1.9 (1.6, 2.4)**	2.6 (0.8, 8.1)	0.7 (0.3, 1.5)	**1.9 (1.3, 2.9)**
LVEF > 50% and cT1 ≥ 800ms (n = 534)	1.0 (0.7, 1.5)	1.1 (0.9, 1.5)	1.1 (0.2, 5.2)	**3.6 (2.3, 5.6)**	**2.3 (1.4, 3.7)**
LVEF ≤ 50% and cT1 < 800ms (n = 550)	**3.0 (2.1, 4.2)**	**1.9 (1.6, 2.4)**	2.6 (0.6, 8.3)	0.7 (0.3, 1.8)	**2.0 (1.2, 3.1)**
LVEF ≤50% and cT1 ≥800ms (n = 54)	**2.6 (1.1, 6.0)**	**2.3 (1.3, 3.9)**	5.0 (0.6, 41)	2.4 (0.6, 10)	**3.5 (1.3, 8.6)**
cT1 (continuous, per s.d.)	1.1 (1.0, 1.2)	**1.1 (1.0, 1.2)**	**1.8 (1.2, 2.6)**	**1.8 (1.6, 2.1)**	**1.4 (1.2, 1.6)**
cT1 800-875ms (n = 502)	1.0 (0.6, 1.5)	1.1 (0.9, 1.5)	0.5 (0.1, 4.3)	**3.2 (2.0, 5.1)**	**2.1 (1.3, 3.3)**
cT1 ≥875ms (n = 86)	1.3, (0.6, 3.0)	1.3 (0.7, 2.3)	**5.2 (1.1, 25.6)**	**5.9 (2.9, 12.2)**	**3.1 (1.2, 7.6)**
Liver fat (continuous, per s.d.)	1.0 (0.9, 1.2)	1.0 (0.9, 1.1)	0.7 (0.4, 1.2)	**1.4 (1.3, 1.6)**	0.9 (0.8, 1.1)
Liver fat 5-10% (n = 1379)	**1.4 (1.1, 1.9)**	1.1 (0.9, 1.4)	0.3 (0.1, 1.4)	**1.7 (1.0, 2.7)**	1.0 (0.7, 1.5)
Liver fat ≥10% (n = 1012)	1.3 (0.9, 1.7)	1.0 (0.8, 1.2)	0.5 (0.1, 1.8)	**3.0 (1.9, 4.7)**	0.8 (0.5, 1.3)

Multivariate adjusted Cox proportional HR (hazard ratio adjusted for age, sex, BMI, type 2 diabetes and dyslipidemia) with 95% confidence intervals are reported. Prevalent CVD defined as recorded diagnosis of hypertension or previous major adverse cardiac event (see supplementary table S2). Statistically significant results (p<0.05) are shown in bold.

Table S9b: Association between MRI metrics of liver health (cT1 and liver fat) and cardiac function (LVEF) and risk of cardiovascular and liver events, hospitalization and all-cause mortality in those without prevalent CVD

	Major CV events	CV-related hospitalisation	Major liver events	Liver-related hospitalisation	All-cause mortality
**Cox proportional hazard ratio (95% CI) (n = 22,149)**					
LVEF ≤50% (n = 921)	**2.1 (1.7, 2.6)**	**2.0 (1.6, 2.5)**	0.7 (0.1, 5.4)	0.8 (0.3, 1.7)	1.0 (0.6, 1.8)
LVEF > 50% and cT1 ≥ 800ms (n = 897)	1.0 (0.7, 1.4)	**1.3 (1.0, 1.8)**	2.9 (0.9, 9.4)	**3.2 (2.1, 5.0)**	1.2 (0.7, 2.0)
LVEF ≤ 50% and cT1 < 800ms (n = 879)	**2.1 (1.7, 2.6)**	**2.0 (1.6, 2.5)**	0.8 (0.1, 6.1)	0.9 (0.4, 2.0)	1.0 (0.6, 1.8)
LVEF ≤50% and cT1 ≥800ms (n = 42)	2.2 (0.8, 5.8)	1.6 (0.5, 4.9)	N.A.	N.A.	N.A.
cT1 (continuous, per s.d.)	**1.1 (1.0, 1.2)**	**1.1 (1.0, 1.2)**	**1.6 (1.1, 2.3)**	**1.4 (1.2, 1.6)**	1.1 (1.0, 1.3)
cT1 800-875ms (n = 795)	1.0 (0.7, 1.4)	**1.4 (1.0, 1.8)**	0.9 (0.1, 6.8)	**2.8 (1.8, 4.5)**	1.3 (0.7, 2.2)
cT1 ≥875ms (n = 144)	0.9 (0.4, 2.0)	0.7 (0.3, 1.7)	**16.0 (3.9, 66.7)**	**4.8 (2.2, 10.5)**	N.A.
Liver fat (continuous, per s.d.)	1.0 (0.9, 1.1)	1.0 (0.9, 1.1)	**1.4 (1.0, 1.8)**	**1.5 (1.3, 1.6)**	1.0 (0.9, 1.2)
Liver fat 5-10% (n = 3329)	0.9 (0.8, 1.1)	1.0 (0.8, 1.2)	0.7 (0.2, 2.6)	**1.8 (1.2, 2.6)**	1.0 (0.7, 1.4)
Liver fat ≥10% (n = 2146)	0.9 (0.7, 1.2)	1.0 (0.8, 1.2)	2.3 (0.8, 6.7)	**3.3 (2.2, 4.9)**	1.3 (0.9, 1.9)

Multivariate adjusted Cox proportional HR (hazard ratio adjusted for age, sex, BMI, type 2 diabetes and dyslipidemia) with 95% confidence intervals are reported. No prevalent CVD defined as excluding those with recorded diagnosis of hypertension or previous major adverse cardiac event (see supplementary table S2). Statistically significant results (p<0.05) are shown in bold.

Table S10a: Association between MRI metrics of liver health (cT1 and liver fat) and cardiac function (LVEF) and risk of cardiovascular and liver events, hospitalization and all-cause mortality in males

	Major CV events	CV-related hospitalisation	Major liver events	Liver-related hospitalisation	All-cause mortality
**Cox proportional hazard ratio (95% CI) (n = 13,571)**					
LVEF ≤50% (n = 1170)	**2.0 (1.6, 2.5)**	**1.9 (1.7, 2.3)**	1.9 (0.7, 5.0)	0.9 (0.5, 1.6)	1.4 (1.0, 2.0)
LVEF > 50% and cT1 ≥ 800ms (n = 803)	1.0 (0.7, 1.3)	1.2 (0.9, 1.5)	1.8 (0.6, 5.6)	**3.4 (2.2, 5.3)**	1.5 (1.0, 2.4)
LVEF ≤ 50% and cT1 < 800ms (n = 1091)	**1.9 (1.6, 2.4)**	**1.9 (1.6, 2.3)**	1.8 (0.6, 5.3)	1.0 (0.5 , 1.9)	**1.5 (1.0, 2.2)**
LVEF ≤50% and cT1 ≥800ms (n = 79)	**2.5 (1.3, 4.7)**	**2.2 (1.3, 3.5)**	4.0 (0.5, 31.3)	2.0 (0.5, 8.3)	1.5 (0.5, 4.7)
cT1 (continuous, per s.d.)	**1.1 (1.0, 1.2)**	**1.1 (1.0, 1.2)**	**1.9 (1.4, 2.6)**	**1.7 (1.5, 2.0)**	**1.2 (1.1, 1.4)**
cT1 800-875ms (n = 766)	0.9 (0.7, 1.3)	1.2 (1.0, 1.5)	0.9 (0.2, 3.9)	**2.9 (1.8, 4.7)**	1.5 (1.0, 2.4)
cT1 ≥875ms (n = 116)	1.4 (0.8, 2.6)	1.4 (0.8, 2.3)	**8.2 (2.2, 31.2)**	**8.3 (4.0, 17.1)**	0.9 (0.2, 3.8)
Liver fat (continuous, per s.d.)	1.0 (0.9, 1.1)	1.0 (0.9, 1.1)	1.1 (0.8, 1.5)	**1.5 (1.3, 1.6)**	0.9 (0.8, 1.1)
Liver fat 5-10% (n = 2838)	1.1 (0.9, 1.3)	1.0 (0.8, 1.2)	0.7 (0.3, 2.1)	**1.7 (1.1, 2.7)**	1.0 (0.8, 1.4)
Liver fat ≥10% (n = 1854)	1.00 (0.8, 1.2)	1.0 (0.8, 1.2)	1.2 (0.4, 3.3)	**3.6 (2.4, 5.6)**	1.0 (0.7, 1.5)

Multivariate adjusted Cox proportional HR (hazard ratio adjusted for age, sex, BMI, type 2 diabetes and dyslipidemia) with 95% confidence intervals are reported

Table S10b: Association between MRI metrics of liver health (cT1 and liver fat) and cardiac function (LVEF) and risk of cardiovascular and liver events, hospitalization and all-cause mortality in females

	Major CV events	CV-related hospitalisation	Major liver events	Liver-related hospitalisation	All-cause mortality
**Cox proportional hazard ratio (95% CI)**					
LVEF ≤50% (n = 355)	**3.7 (2.7, 5.1)**	**2.8 (2.0, 3.8)**	N.A	0.2 (0.0, 1.6)	1.8 (0.9, 3.9)
LVEF > 50% and cT1 ≥ 800ms (n = 628)	1.2 (0.7, 1.8)	1.4 (1.0, 2.0)	2.4 (0.5, 12.6)	**3.5 (2.3, 5.3)**	**2.1 (1.1, 3.9)**
LVEF ≤ 50% and cT1 < 800ms (n = 328)	**3.8 (2.7, 5.3)**	**2.9 (2.1, 4.1)**	N.A	0.3 (0.0, 2.1)	1.4 (0.6, 3.5)
LVEF ≤50% and cT1 ≥800ms (n = 17)	**N**/A	**N/A**	N.A	N.A	**17.9 (4.3, 74.7)**
cT1 (continuous, per s.d.)	1.1 (1.0, 1.2)	**1.1 (1.0, 1.2)**	1.5 (0.9, 2.3)	**1.4 (1.3, 1.7)**	**1.3 (1.1, 1.5)**
cT1 800-875ms (n = 531)	1.2 (0.8, 1.9)	**1.5 (1.0, 2.1)**	N.A	**3.4 (2.1, 5.3)**	**2.2 (1.2, 4.2)**
cT1 ≥875ms (n = 114)	0.3 (0.0, 2.0)	0.4 (0.1, 1.6)	**15.4 (2.9, 81.2)**	**3.9 (1.8, 8.5)**	3.0 (0.9, 9.6)
Liver fat (continuous, per s.d.)	1.0 (0.9, 1.1)	1.0 (0.9, 1.1)	1.1 (1.7, 1.8)	**1.4 (1.3, 1.6)**	1.0 (0.9,1.3)
Liver fat 5-10% (n = 1870)	1.0 (0.8, 1.4)	1.1 (0.9, 1.4)	0.6 (0.1, 2.4)	**1.8 (1.2., 2.8)**	1.0 (0.6, 1.6)
Liver fat ≥10% (n = 1304)	1.1 (0.8, 1.5)	1.0 (0.7, 1.3)	N.A.	**2.9 (1.9, 4.4)**	1.2 (0.7, 2.1)

Multivariate adjusted Cox proportional HR (hazard ratio adjusted for age, sex, BMI, type 2 diabetes and dyslipidemia) with 95% confidence intervals are reported. In the female cohort (n=15,270) there are relatively few liver events (n=16). Expanding the female cohort to include participants with available cT1 and LFC regardless of available CMR metrics (n=17,666) increases the number of liver events to 21. In this expanded cohort, continuous cT1 is associated with major liver events (adjusted HR 1.6 (1.1, 2.4)) but LFC is not (1.2 (0.8, 1.7)), following the same pattern seen in male participants.

Supplementary Figure 1a: Forrest plot of hazard ratios by cT1 and ejection fraction category

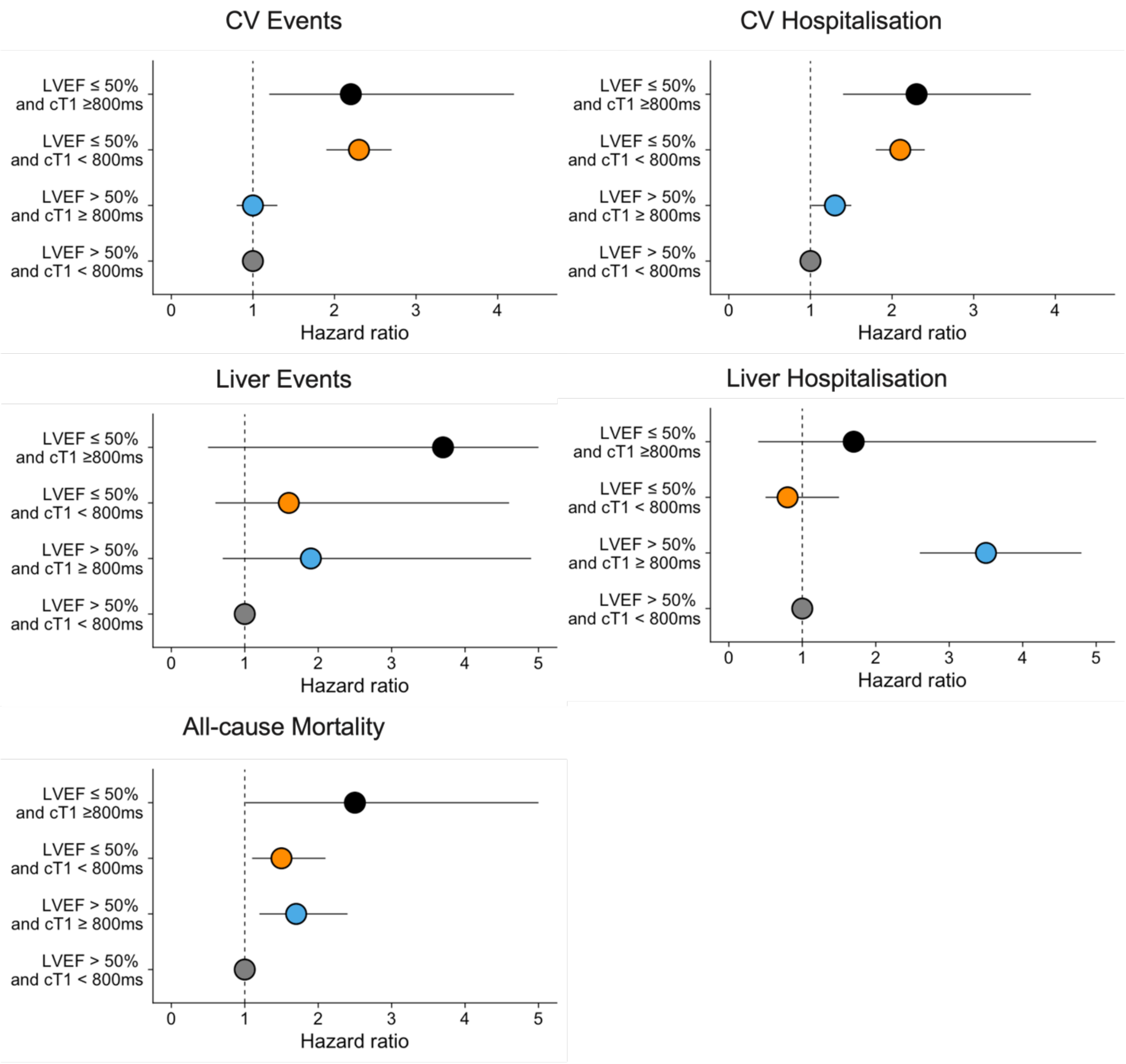

HR (Cox proportional hazard ratio adjusted for age, sex, BMI, type 2 diabetes and dyslipidemia) with 95% confidence intervals are shown.

Supplementary Figure 1b: Forrest plot of hazard ratios by cT1 threshold

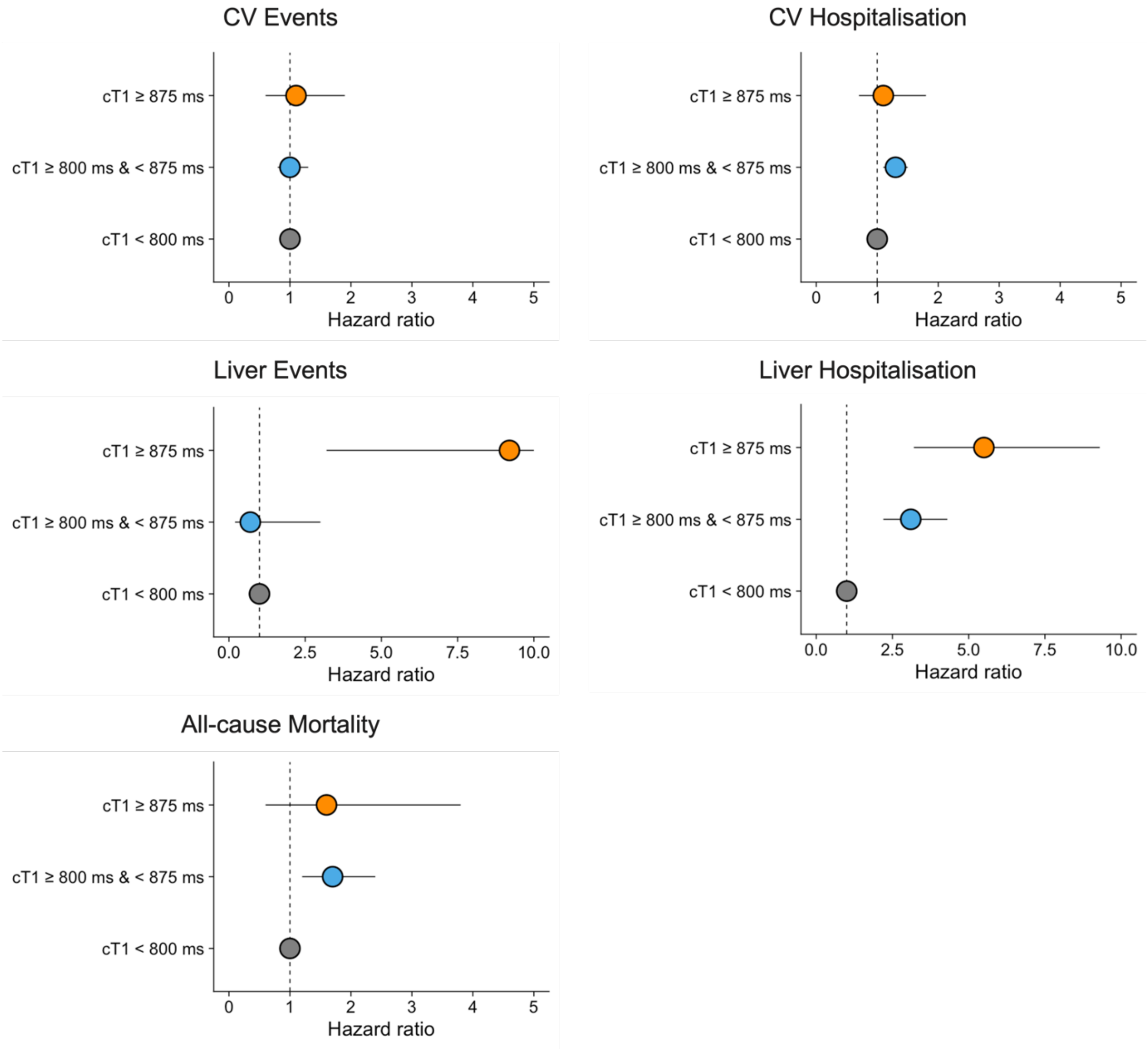

HR (Cox proportional hazard ratio adjusted for age, sex, BMI, type 2 diabetes and dyslipidemia) with 95% confidence intervals are shown.

References

1. Bosco E, Hsueh L, McConeghy KW, Gravenstein S, Saade E. Major adverse cardiovascular event definitions used in observational analysis of administrative databases: a systematic review. BMC Med Res Methodol. 2021 Dec 6;21(1):241.
2. Roca-Fernandez A, Banerjee R, Thomaides-Brears H, Telford A, Sanyal A, Neubauer S, et al. Liver disease is a significant risk factor for cardiovascular outcomes - a UK Biobank study. J Hepatol. 2023 Jun;
3. Shang Y, Akbari C, Dodd M, Zhang X, Wang T, Jemielita T, et al. Association between longitudinal biomarkers and major adverse liver outcomes in patients with

non-cirrhotic metabolic dysfunction–associated steatotic liver disease. Hepatology. 2024 Aug 7;
4. Ito S, Miranda WR, Nkomo VT, Connolly HM, Pislaru S V., Greason KL, et al. Reduced Left Ventricular Ejection Fraction in Patients With Aortic Stenosis. J Am Coll Cardiol. 2018 Mar;71(12):1313–21.
5. Andersson A, Kelly M, Imajo K, Nakajima A, Jonathan A, Hirschfield G, et al. Clinical utility of MRI biomarkers for identifying NASH patients at high risk of progression: A multi-center pooled data and meta-analysis. Clinical Gastroenterology and Hepatology [Internet]. 2021; Available from: https://doi.org/10.1016/j.cgh.2021.09.041
6. Tacke F, Horn P, Wai-Sun Wong V, Ratziu V, Bugianesi E, Francque S, et al. EASL–EASD–EASO Clinical Practice Guidelines on the management of metabolic dysfunction-associated steatotic liver disease (MASLD). J Hepatol. 2024 Sep;81(3):492–542.
7. Andersson A, Kelly M, Imajo K, Nakajima A, Fallowfield JA, Hirschfield G, et al. Clinical Utility of Magnetic Resonance Imaging Biomarkers for Identifying Nonalcoholic Steatohepatitis Patients at High Risk of Progression: A Multicenter Pooled Data and Meta-Analysis. Vol. 20, Clinical Gastroenterology and Hepatology. W.B. Saunders; 2022. p. 2451-2461.e3.
8. Bachtiar V, Wilman H, Jacobs J, Newbould R, Kelly C, Gyngell M, et al. Reliability and reproducibility of multiparametric magnetic resonance imaging of the liver. PLoS One. 2019;14(4):e0214921.
9. Fowler KJ, Venkatesh SK, Obuchowski N, Middleton MS, Chen J, Pepin K, et al. Repeatability of MRI Biomarkers in Nonalcoholic Fatty Liver Disease: The NIMBLE Consortium. Radiology. 2023 Oct 1;309(1).
