# Supplementary material for: Cardiac and liver impairment on multiorgan MRI and risk of major adverse cardiovascular and liver events
Source: Nat Med. 2025 May 7;31(7):2289–96. doi: 10.1038/s41591-025-03654-2 (PMC12283365; doi:10.1038/s41591-025-03654-2)
Supplement: Supplementary file 2 — Reporting Summary [file 41591_2025_3654_MOESM2_ESM.pdf]

nature portfolio

Corresponding author(s): Amitava Banerjee

Last updated by author(s): Mar 3, 2025

Reporting Summary

Nature Portfolio wishes to improve the reproducibility of the work that we publish. This form provides structure for consistency and transparency in reporting. For further information on Nature Portfolio policies, see our Editorial Policies and the Editorial Policy Checklist.

Statistics

For all statistical analyses, confirm that the following items are present in the figure legend, table legend, main text, or Methods section.

n/a	Confirmed	
☐	☒	The exact sample size (*n*) for each experimental group/condition, given as a discrete number and unit of measurement
☐	☒	A statement on whether measurements were taken from distinct samples or whether the same sample was measured repeatedly
☐	☒	The statistical test(s) used AND whether they are one- or two-sided<br>*Only common tests should be described solely by name; describe more complex techniques in the Methods section.*
☐	☒	A description of all covariates tested
☐	☒	A description of any assumptions or corrections, such as tests of normality and adjustment for multiple comparisons
☐	☒	A full description of the statistical parameters including central tendency (e.g. means) or other basic estimates (e.g. regression coefficient) AND variation (e.g. standard deviation) or associated estimates of uncertainty (e.g. confidence intervals)
☐	☒	For null hypothesis testing, the test statistic (e.g. *F*, *t*, *r*) with confidence intervals, effect sizes, degrees of freedom and *P* value noted<br>*Give P values as exact values whenever suitable.*
☒	☐	For Bayesian analysis, information on the choice of priors and Markov chain Monte Carlo settings
☒	☐	For hierarchical and complex designs, identification of the appropriate level for tests and full reporting of outcomes
☒	☐	Estimates of effect sizes (e.g. Cohen's *d*, Pearson's *r*), indicating how they were calculated

Our web collection on statistics for biologists contains articles on many of the points above.

Software and code

Policy information about availability of computer code

Data collection	No software was used to collect the data.
Data analysis	RStudio 2024.12.0+467, r version 4.0.4

For manuscripts utilizing custom algorithms or software that are central to the research but not yet described in published literature, software must be made available to editors and reviewers. We strongly encourage code deposition in a community repository (e.g. GitHub). See the Nature Portfolio guidelines for submitting code & software for further information.

Data

Policy information about availability of data

All manuscripts must include a data availability statement. This statement should provide the following information, where applicable:

- Accession codes, unique identifiers, or web links for publicly available datasets
- A description of any restrictions on data availability
- For clinical datasets or third party data, please ensure that the statement adheres to our policy

All data in this study is from the UK Biobank (UKB). UKB data is made available to researchers from research institutions with genuine research inquiries, following IRB and UKB approval. Requests to access these datasets should be directed to access@ukbiobank.ac.uk.

Research involving human participants, their data, or biological material

Policy information about studies with human participants or human data. See also policy information about sex, gender (identity/presentation), and sexual orientation and race, ethnicity and racism.

Reporting on sex and gender	Sex was used in this study based on UKBB data field 31. The definition of field 31 is "[Sex] ...acquired from central registry at recruitment, but in some cases updated by the participant. Hence this field may contain a mixture of the sex the NHS had recorded for the participant and self-reported sex." Sex has been used as a covariate in the main text and results disaggregated for sex are shown in the supplementary materials.
Reporting on race, ethnicity, or other socially relevant groupings	Ethnicity (% white British) is reported in this analysis.
Population characteristics	A full description of the study population's characteristics is given in table 1 of the manuscript
Recruitment	The UK Biobank cohort is a population-based cohort of 500,000 participants recruited in the United Kingdom (UK) between 2006 and 2010. Approximately 9.2 million individuals aged 40–69 years who lived within 25 miles (40 km) of one of 22 assessment centers in England, Wales, and Scotland were invited to enter the cohort, and 5.5% participated in the baseline assessment. A subset of this population volunteered to be part of the imaging sub-study.
Ethics oversight	UKB data use (project application no. 9914) was approved by the UKB according to their established access procedures. UK Biobank has approval from the North West Multi-centre Research Ethics Committee (MREC) as a Research Tissue Bank (RTB) approval. This approval means that researchers do not require separate ethical clearance and can operate under the RTB approval.

Note that full information on the approval of the study protocol must also be provided in the manuscript.

Field-specific reporting

Please select the one below that is the best fit for your research. If you are not sure, read the appropriate sections before making your selection.

☒ Life sciences ☐ Behavioural & social sciences ☐ Ecological, evolutionary & environmental sciences

For a reference copy of the document with all sections, see nature.com/documents/nr-reporting-summary-flat.pdf

Life sciences study design

All studies must disclose on these points even when the disclosure is negative.

Sample size	This paper is an observational analysis of the association between imaging metrics and outcomes in the UK biobank. As such, sample size calculations were not performed in advance but all available participants were analysed.
Data exclusions	Participants with incomplete imaging metrics were excluded from the analysis. For analysis of new-onset outcomes, prevalent disease is excluded where specified in the text..
Replication	It is not currently possible to to replicate this study as the only large scale biobank with this data available is the UK biobank. However, future studies in targeted high risk popultaions and other large scale biobanks that will be available in the future are mentioned in the discussion as ways of replicating these analyses.
Randomization	As there is no treatment arm, participants were not randomised. Participants were separated into different categories based on imaging phenotypes with previously calculated clinically relevant cutoffs.
Blinding	This was a prospective observational study using data independently available from the UKB and no blinding was performed.

Reporting for specific materials, systems and methods

We require information from authors about some types of materials, experimental systems and methods used in many studies. Here, indicate whether each material, system or method listed is relevant to your study. If you are not sure if a list item applies to your research, read the appropriate section before selecting a response.

Materials & experimental systems

n/a	Involved in the study	
☒	☐	Antibodies
☒	☐	Eukaryotic cell lines
☒	☐	Palaeontology and archaeology
☒	☐	Animals and other organisms
☐	☒	Clinical data
☒	☐	Dual use research of concern
☒	☐	Plants

Methods

n/a	Involved in the study	
☒	☐	ChIP-seq
☒	☐	Flow cytometry
☒	☐	MRI-based neuroimaging

Clinical data

Policy information about clinical studies

All manuscripts should comply with the ICMJE guidelines for publication of clinical research and a completed CONSORT checklist must be included with all submissions.

Clinical trial registration	UK Biobank has approval from the North West Multi-centre Research Ethics Committee (MREC) as a Research Tissue Bank (RTB) approval. This approval means that researchers do not require separate ethical clearance and can operate under the RTB approval.
Study protocol	As UK biobank sub study, once a UK biobank application is approved, researchers do not require separate ethical clearance and can operate under the RTB approval.
Data collection	Imaging data was acquired from 2014 onwards. Clinical trial data was collected until October 2022.
Outcomes	Outcomes are major liver and cardiovascular events, and all-cause mortality. These are collected from self reported data, primary healthcare data, hospital inpatient data and death records, collected by the UK biobank.

Plants

Seed stocks	*Report on the source of all seed stocks or other plant material used. If applicable, state the seed stock centre and catalogue number. If plant specimens were collected from the field, describe the collection location, date and sampling procedures.*
Novel plant genotypes	*Describe the methods by which all novel plant genotypes were produced. This includes those generated by transgenic approaches, gene editing, chemical/radiation-based mutagenesis and hybridization. For transgenic lines, describe the transformation method, the number of independent lines analyzed and the generation upon which experiments were performed. For gene-edited lines, describe the editor used, the endogenous sequence targeted for editing, the targeting guide RNA sequence (if applicable) and how the editor was applied.*
Authentication	*Describe any authentication procedures for each seed stock used or novel genotype generated. Describe any experiments used to assess the effect of a mutation and, where applicable, how potential secondary effects (e.g. second site T-DNA insertions, mosiacism, off-target gene editing) were examined.*
